# Utilization of Rosuvastatin and Endogenous Biomarkers in Evaluating the Impact of Ritlecitinib on BCRP, OATP1B1, and OAT3 Transporter Activity

**DOI:** 10.1007/s11095-023-03564-3

**Published:** 2023-08-10

**Authors:** Yeamin Huh, Anna Plotka, Hua Wei, Julia Kaplan, Nancy Raha, Justin Towner, Vivek S. Purohit, Martin E. Dowty, Robert Wolk, Manoli Vourvahis, Amanda King-Ahmad, Sumathy Mathialagan, Mark A. West, Sarah Lazzaro, Sangwoo Ryu, A. David Rodrigues

**Affiliations:** 1 Pfizer Inc, Groton, CT USA; 2 Pfizer Inc, Collegeville, PA USA; 3 Pfizer Inc, Shanghai, China; 4 Pfizer Inc, Cambridge, MA USA; 5 Pfizer Inc, New York, NY USA

**Keywords:** drug-drug interaction, endogenous biomarker, ritlecitinib, rosuvastatin

## Abstract

**Purpose:**

Ritlecitinib, an inhibitor of Janus kinase 3 and tyrosine kinase expressed in hepatocellular carcinoma family kinases, is in development for inflammatory diseases. This study assessed the impact of ritlecitinib on drug transporters using a probe drug and endogenous biomarkers.

**Methods:**

*In vitro* transporter-mediated substrate uptake and inhibition by ritlecitinib and its major metabolite were evaluated. Subsequently, a clinical drug interaction study was conducted in 12 healthy adult participants to assess the effect of ritlecitinib on pharmacokinetics of rosuvastatin, a substrate of breast cancer resistance protein (BCRP), organic anion transporting polypeptide 1B1 (OATP1B1), and organic anion transporter 3 (OAT3). Plasma concentrations of coproporphyrin I (CP-I) and pyridoxic acid (PDA) were assessed as endogenous biomarkers for OATP1B1 and OAT1/3 function, respectively.

**Results:**

*In vitro* studies suggested that ritlecitinib can potentially inhibit BCRP, OATP1B1 and OAT1/3 based on regulatory cutoffs. In the subsequent clinical study, coadministration of ritlecitinib decreased rosuvastatin plasma exposure area under the curve from time 0 to infinity (AUC_inf_) by  ~ 13% and maximum concentration (C_max_) by  ~ 27% relative to rosuvastatin administered alone. Renal clearance was comparable in the absence and presence of ritlecitinib coadministration. PK parameters of AUC_inf_ and C_max_ for CP-I and PDA were also similar regardless of ritlecitinib coadministration.

**Conclusion:**

Ritlecitinib does not inhibit BCRP, OATP1B1, and OAT3 and is unlikely to cause a clinically relevant interaction through these transporters. Furthermore, our findings add to the body of evidence supporting the utility of CP-I and PDA as endogenous biomarkers for assessment of OATP1B1 and OAT1/3 transporter activity.

**Supplementary Information:**

The online version contains supplementary material available at 10.1007/s11095-023-03564-3.

## Introduction

Transporters are expressed in various tissues and play an important role in the absorption, distribution and excretion of drugs and endogenous molecules [1, 2]. Understanding of drug-drug interaction (DDI) via transporters is important in drug development given the high likelihood of concomitant use of multiple medications. In line with this, *in vitro* assessment of transporter-mediated DDI and subsequent clinical DDI studies becomes standard practice during drug development to guide the prohibited/permitted concomitant medications for clinical studies in patients and eventually to inform product labeling.

Ritlecitinib is an oral, covalent inhibitor of Janus kinase (JAK) 3 and the tyrosine kinase expressed in hepatocellular carcinoma (TEC) family kinases [3, 4]. Treatment with ritlecitinib is expected to inhibit the inflammatory pathways mediated by interleukin (IL)-7, IL-15 and IL-21, which have been implicated in the pathogenic pathways of alopecia areata, vitiligo, inflammatory bowel disease, and rheumatoid arthritis. Therefore, ritlecitinib is in development for various autoimmune and inflammatory diseases and has recently shown positive efficacy and safety data in a pivotal phase 2b/3 trial in alopecia areata [5]. Given that patients to be treated with ritlecitinib may have medical comorbidities that require treatment with other medications, understanding of transporter-mediated DDI risk is important [6].

However, challenges exist when studying transporter-mediated DDI. The current regulatory thresholds of *in vitro* DDI risk may trigger unnecessary clinical DDI studies which demonstrate false-positiveness of the *in vitro* predictions, with healthy participants unnecessarily exposed to drugs. Additionally, probe drugs supporting clinical DDI studies are often substrates of multiple transporters, which limits the mechanistic interpretation of DDI observations and extrapolation of the results to other potential concomitant medications [7]. In order to overcome such challenges, various endogenous transporter substrates have been studied as plasma- and urine-based biomarkers that can be readily deployed in Phase 1 studies to obviate a need for formal (drug probe-based) clinical DDI studies. This has been showcased by recent reports describing the successful use of endogenous biomarkers to elucidate the mechanism of complex DDI and de-risk DDI potential via individual transporters [8, 9].

In a recent study, inhibition of hepatic organic cation transporter (OCT) 1 by ritlecitinib was evaluated using sumatriptan as a probe substrate drug [10]. In the same study, the endogenous biomarker of N^1^-methylnicotinamide (NMN) was successfully used to de-risk the inhibition of renal multidrug and toxin extrusion proteins (MATE1 and MATE2K) and organic cation transporter (OCT) 2 by ritlecitinib, based on the lack of changes in NMN renal clearance with ritlecitinib coadministration. At the same time, the clinical utility of plasma isobutyryl-L-carnitine (IBC) as an endogenous biomarker of liver OCT1 was also demonstrated during the study of ritlecitinib and its major inactive circulating metabolite (cysteine conjugate of ritlecitinib, M2) as OCT1 inhibitors.

In this study, another transporter-mediated DDI risk assessment for ritlecitinib is presented using both a clinical probe and endogenous biomarkers. *In vitro* studies presented herein suggested that ritlecitinib can potentially inhibit breast cancer resistance protein (BCRP), organic anion transporting polypeptide 1B1 (OATP1B1), and organic anion transporter (OAT)1/3 based on their relevant regulatory thresholds. As a subsequent step, a dedicated clinical DDI study was conducted to investigate the effect of ritlecitinib on these transporters. Therefore, the purpose of the current study is to assess the effect of ritlecitinib on the *in vivo* pharmacokinetics (PK) of rosuvastatin, as a BCRP, OATP1B1, and OAT3 substrate. Plasma-based endogenous biomarkers of coproporphyrin I (CP-I) and pyridoxic acid (PDA) were used as endogenous biomarkers to separately evaluate the possible impact of OATP1B1 and OAT3 inhibition, respectively, on changes in rosuvastatin systemic exposure and renal clearance by ritlecitinib. As described herein, it was possible to expand beyond NMN and IBC to include two additional plasma-based biomarkers, CP-I and PDA, to evaluate transporter-mediated DDI risk of ritlecitinib via OATP1B1 and OAT3.

## Materials and Methods

### *In Vitro* Transporter-Mediated Substrate Uptake and Inhibition

Sources of human embryonic kidney (HEK) 293 cells expressing individual transfected human solute carriers (SLC), test substrates, and cell culture reagents, as well as detailed cell culture methods, incubations, and sample processing, have been described previously [11–13]. SLC included hepatic OATP1B1, OATP1B3, and renal OAT1 and OAT3. Uptake of radiolabeled [^3^H]*p*-aminohippuric acid (^3^H-PAH, PerkinElmer, Waltham, MA) by OAT1 at 4 min and [^3^H]estrone 3-sulfate (^3^H-E3S, PerkinElmer, Waltham, MA) by OAT3 at 3 min was measured at a final concentration of 0.5 and 0.2 µM, respectively (< the reported Michaelis constant K_m_) [14]. Likewise, the uptake of non-labeled rosuvastatin (Biosynth, San Diego, CA) at 0.3—0.5 µM was determined for OATP1B1 and OATP1B3 at 1—3 min, and CP-I (Frontier Specialty Chemicals, Logan, UT) at 0.1 µM was determined for OATP1B1 and OATP1B3 at 10 min [14].

As described previously, BCRP inhibition by ritlecitinib was measured using BCRP-expressing membrane vesicles with non-labeled rosuvastatin as substrate [15]. The concentration of RSV (0.2 μM) used in these studies was below its BCRP K_m_ (3.2 μM).

The IC_50_ (concentration of test inhibitor presenting 50% inhibition of uptake) was determined for each substrate. In all cases, ritlecitinib (0.018 to 1000 µM) and metabolite M2 (0.018 to 300 µM) was tested over a wide range of final concentrations dissolved in dimethyl sulfoxide (the final concentration of dimethyl sulfoxide in the assays was 1% v/v). For each transporter, the IC_50_ was estimated using a four-parameter logistic equation (Eq. 1) using GraphPad Prism software (GraphPad Software Inc, La Jolla, CA).

1 $$\%\;Uptake\;Activity=Bottom+\frac{(Top-Bottom)}{1+10^{(\left(Log{IC}_{50}-\left[inhib\right]\right)\ast HillSlope)}}$$

### Bioanalysis of *In Vitro* Study Samples

At the end of the uptake and inhibition experiment, cellular accumulation of ^3^H-PAH and ^3^H-E3S was determined using liquid scintillation counting as previously described [11–13]. Rosuvastatin uptake was determined using a tandem high performance liquid chromatography (LC)-mass spectrometry (MS) assay [12].

Uptake of CP-I was determined by analysis of the *in vitro* assay samples using an optimized LC-high resolution mass spectrometry (HRMS) method. In brief, dried samples were reconstituted in 150 µL of 0.1% formic acid and analyzed by LC-HRMS. Analyte separation and detection was achieved using a Shimadzu Nexera high performance LC system (Shimazdu Scientific, MA, USA) coupled to a Sciex API-6600 Q-TOF (time of flight) mass spectrometer with a Turbo IonDrive source (Sciex Corporation, MA, USA). Chromatographic separation was achieved using a Waters Acquity BEH C18 1.7µ 2.1 mm x 100 mm column and a binary mobile phase system consisting of 0.1% v/v formic acid and 0.1% v/v formic acid in acetonitrile, as mobile phase A and B, respectively. The column temperature was held at 60 °C and the flow rate was 0.55 mL/minute. The gradient program had initial conditions of 5% B which was held for 0.9 min, followed by a linear ramp to 95% B over 5.1 min, a hold for 1.0 min, and a return to starting conditions over 0.1 min with re-equilibration for 0.9 min (total run time of 8.0 min). Analyte detection was achieved using an API-6600 Q T-OF mass spectrometer with electrospray ionization (ESI). The mass spectrometer was operated in both TOF-MS and TOF-MS/MS mode with data collected over a 60 to 670 dalton mass range. TOF-MS/MS data were collected in the high-resolution mode. Quantification of CP-I levels was assessed using targeted TOF-MS/MS data with accumulation times of 150 and 35 ms, respectively, for CP-I and the internal standard (ISTD) verapamil. Data were processed using the MultiQuant software package using the targeted mass-to-charge transitions of 328.1 to 268.1201 ± 0.02 daltons and 238.1094 ± 0.02 daltons (summed ions) for CP-I, and 455.2 to 165.0907 ± 0.02 daltons for the ISTD (verapamil), to generate analyte/ISTD area ratios.

### Clinical Study Design

**Fig. 1:**
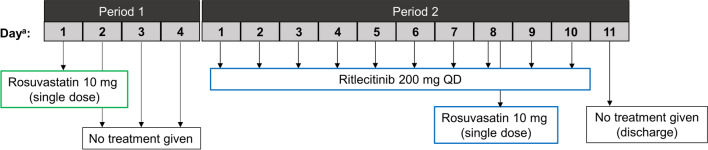
Treatment flow diagram. *Day was relative to the first day of study medicine dosing in each period. Day 1 of Period 2 was the same as Day 5 of Period 1.

This was a Phase 1, 2-period fixed-sequence, multiple-dose, open-label study of the effect of ritlecitinib on the pharmacokinetics and renal clearance of a single, oral dose of rosuvastatin in healthy participants (Fig. 1). The safety and tolerability of rosuvastatin when coadministered with ritlecitinib was also evaluated.

#### Study Participants

Eligible study participants were healthy men and women aged 18 to 55 years, with a body mass index of 17.5 to 30.5 kg/m^2^ and a total body weight  > 50 kg (110 lb). Use of prescription or nonprescription drugs and dietary supplements within 7 days or 5 half-lives (whichever is longer) prior to Day 1 was prohibited. Limited use of nonprescription medications that are not expected to affect participant safety or overall study results was permitted on a case-by-case basis. All participants provided signed informed consent.

#### Treatments

Participants remained in the Pfizer Clinical Research Unit (PCRU), New Haven, CT, USA, for a total of 16 days and 15 nights: 5 days and 5 nights in Period 1, and 11 days and 10 nights in Period 2 (Fig. 1). In Period 1 on Day 1, following an overnight fast of at least 10 h, participants received a single oral administration of rosuvastatin 10 mg tablet. Period 1 was immediately followed by Period 2 with no washout. In Period 2 (coadministration), participants received oral ritlecitinib 200 mg once daily (QD) under non-fasting conditions for 7 days. On the morning of Day 8, a single dose of rosuvastatin 10 mg tablet was administered orally within approximately 5 min after administration of a ritlecitinib 200 mg dose under fasting conditions. On Days 9 and 10, participants continued to receive oral ritlecitinib 200 mg QD under non-fasting conditions.

Drugs were administered with approximately 240 mL of ambient temperature water during Period 1; during Period 2, participants could receive additional ambient temperature water up to 100 mL, if needed. Ritlecitinib 200 mg was provided as 4 × 50 mg tablets and rosuvastatin as a 1 × 10 mg tablet.

#### Blood and Urine Sample Collection

Blood and urine samples were collected at prespecified timepoints. Rosuvastatin plasma samples (~ 6 mL) were collected predose and at 0.5, 1, 2, 3, 4, 5, 6, 8, 10, 12, 16, 24, 36, 48, and 72 h postdose. CP-I and PDA plasma samples (~ 6 mL) were collected predose and at 0.5, 1, 2, 3, 4, 5, 6, 8, 10, 12, 16, and 24 h post rosuvastatin dosing. Rosuvastatin urine samples were collected at predose, 0–6 h, 6–12, 12–24, 24–48, and 48–72 h postdose. Each participant emptied their bladder just prior to dosing.

#### Analytical Methods for Rosuvastatin in Plasma

Plasma rosuvastatin was measured using a validated UPLC-MS/MS assay, in compliance with Pfizer standard operating procedures. Samples were thawed at room temperature and vortexed. After addition of internal standard working solution to a 0.100 mL aliquot of the samples, rosuvastatin and its stable isotope labeled internal standard rosuvastatin-d3 were isolated from human plasma using a liquid-liquid extraction procedure. During the procedure 1000 µL of 0.1N hydrochloric acid was added to the tube, vortexed, followed by addition of 5 mL of methyl tret-butyl ether (MTBE), and shaken on horizontal shaker at 240 excursions/minute for 15 min; and then centrifuged for 5 min at 3000 rpm, at 4 °C. Samples were then placed in a methanol/ dry ice bath for 5 min. The organic phase was transferred into conical 16 × 100 mm borosilicate culture tubes, and the organic phase was evaporated to dryness under 15 psi of nitrogen (about 15 min) at 50ºC on Turbo Vap evaporator. Dry residues were reconstituted with 200 μL of reconstitution solution (mobile phase A). After vortexing, all tubes were centrifuged at 3000 rpm for 5 min at 4 °C, and 150 μL of the reconstituted samples were transferred into the deep round well microplates and sealed by aluminum foil at 165ºC. Reconstituted extracts were analyzed by UPLC-MS/MS using an ACE 3 C18, 30 × 3.0 mm, 3 µm column and API 5000 detector with TurboIonSpray® in positive mode. Mobile phase A was Milli-Q Type Water/methanol, ammonium acetate 2 mM and mobile phase B was acetonitrile. The monitored ion transitions were m/z 483.3 → 258.2; (RT = 1.54 min) for rosuvastatin and m/z 485.3 → 261.2 (RT = 1.51) for the internal standard (rosuvastatin-d3). The validated rosuvastatin concentration calibration range was 20 to 25000 pg/mL.

Interrun accuracy (percentage relative error) across the study ranged from  − 10.72% to  − 2.13%, and interrun precision (percentage coefficient of variation) was  ≤ 2.56%.

#### Analytical Methods for Rosuvastatin in Urine

Urine rosuvastatin was measured using a validated HPLC-MS/MS assay, in compliance with Pfizer standard operating procedures. Rosuvastatin was extracted from an aliquot of 0.050 mL of human urine using an automated liquid-liquid extraction procedure. The final extract was then analyzed by HPLC-MS/MS using an ACE 3 C18, 30 × 3.0 mm, 3 µm column and API 4000 detector with a Turbo Ionspray® interface. Mobile phase A was Milli-Q® type water/methanol, ammonium acetate 2 mM and mobile phase B was acetonitrile. The monitored ion transitions were m/z 483.3 → 258.2; (RT = 1.21 min) for rosuvastatin and m/z 485.3 → 261.2 (RT = 1.19) for the internal standard (rosuvastatin-d3). The validated rosuvastatin concentration calibration range was 5 to 2000 ng/mL.

Interrun accuracy (percentage relative error) across the study ranged from 0.73% to 5.28%, and interrun precision (percentage coefficient of variation) was  ≤ 14.95%.

#### Analytical Methods for Endogenous Biomarkers

Quantification of CP-I was conducted by mass spectrometry according to King-Ahmad *et al*. [16]. Plasma samples were spiked with stable label internal standard (^15^N_4_-coproporphyrin I). CP-I was isolated through supported liquid extraction. The eluate was evaporated under a nitrogen stream at approximately 45 °C, and the remaining residue was reconstituted with 125 μL of water/acetonitrile/formic acid (750:250: 1, v/v/v). The final extract was analyzed via UPLC® with column switching and MS/MS detection using positive ion electrospray. A linear, 1/x^2^ weighted, least-squares regression algorithm was used to quantitate sample concentrations.

Quantification of PDA was conducted by mass spectrometry according to Towner *et al*. [17]. All separations of analytes were done on an Acquity UPLC system and were achieved at 40 °C using a Waters Atlantis HILIC silica column, 2.1 × 100 mm, 3.0 μm under isocratic conditions of 5% (acetonitrile containing 0.1% formic acid [mobile phase A]) and 95% (95:5 acetonitrile containing 0.1% formic acid:10 mM ammonium formate in water containing 0.1% formic acid [mobile phase B]) at a flow rate of 0.5 mL/min for a total run time of 3 min. Mass spectrometry detection of the analytes were achieved using a SCIEX API QTrap 5500 mass spectrometry and a Turbo V electrospray ionization source in negative ion mode. Plasma samples were prepared by protein precipitation by spiking 50 μL of sample with 200 μL of 300 ng/mL 4-aminosalicylic acid internal standard. After vortexing/centrifugation, 200 μL of supernatant was transferred and 1 μL injections were analyzed. A 1/x^2^ weighted linear regression was used to quantitate sample concentrations.

#### Genotyping

A whole blood sample (~ 4 mL) was collected from each participant to perform genotyping for 2 SNPs for ABCG2 (gene encoding for BCRP), rs2231142 (421C > A) and rs72552713 (376C > T) and for 2 SNPs for SLCO1B1 (gene encoding OATP1B1), rs2306283 (*1B, 388A > G) and rs4149056 (*5, 521 T > C). All 4 SNPs were genotyped using commercially available TaqMan® assays and analyzed on a QuantStudio 12 K Flex Real-Time PCR System.

#### Statistical Methods and PK Parameters

The PK parameters for rosuvastatin, CP-I and PDA were calculated for each participant and each treatment using Pfizer’s internally validated electronic noncompartmental analysis software (eNCA) version 2.4.4. Samples below the lower limit of quantitation were set to zero for the PK analysis. Actual sample collection times were used for the PK analysis.

Natural log transformed area under the plasma concentration-time profile from time 0 extrapolated to infinity (AUC_inf_), maximum plasma concentration (C_max_), and apparent renal clearance (CL_r_) were analyzed using a mixed effect model with treatment as a fixed effect and participant as a random effect (SAS version 9.4; SAS Institute, Cary, NC, USA). Estimates of the adjusted mean differences (Test-Reference) and corresponding 90% CIs were obtained from the model. The adjusted mean difference and 90% CIs for the differences were exponentiated to provide estimates of the ratio of adjusted geometric means (Test/Reference) and 90% CIs for the ratios. Rosuvastatin alone was the reference treatment, while the rosuvastatin coadministered with ritlecitinib was the Test treatment.

## Results

### *In Vitro* Data and the Prediction of Biomarker Response

Ritlecitinib OATP1B1 and OATP1B3 IC_50_ data, with CP-I and rosuvastatin as substrates, as well as OAT1 (PAH as substrate) and OAT3 (E3S as substrate) IC_50_s are shown in Table I. For both OATP1B1 and OATP1B3, the IC_50_ values for CP-I and RSV were similar and no substrate-dependency was evident. In the case of OAT3 and OAT1, the IC_50_ values were lower (41 and 156 µM, respectively) *versus* OATP1B1/3. Overall, the estimated fraction inhibited *in vivo* was low (≤ 0.1) for all four transporters, although ritlecitinib met the European Medicines Agency (EMA) drug interaction risk cutoff for OAT1 and OAT3, as well as the EMA and US Food & Drug Administration cutoff for OATP1B1 (Table I). Ritlecitinib also inhibited BCRP (IC_50_ = 27 µM), presented a *G*-value (ratio of estimated concentration in the gut [I]_2_/IC_50_ ratio) of 104, and exceeded its drug interaction risk cutoff (Table I). To complete the drug interaction risk assessment, the major circulating metabolite (M2) was also tested *in vitro* as an OAT1/3 and OATP1B1/3 inhibitor. Only weak inhibition of the former (IC_50_ > 300 µM) was evident. In comparison, M2 presented inhibition of both OATP1B1 (IC_50_ = 2.0 µM) and OATP1B3 (IC_50_ = 8.4 µM) and exceeded the drug interaction risk cutoff for both transporters like parent ritlecitinib.

**Table I:** Ritlecitinib and its Metabolite (M2) as in Vitro Inhibitors of Drug Transporters

Transporter (Substrate)^a^	IC_50_ (K_i_) (μM)^b^	[I]_2_ (μM)^c^	C_max,u_ (μM)^c^	l_max,inlet,u_ (μM)^d^	*R*-value^e^	[I]_2_/IC_50_ or C_max,u_/IC_50_ Ratio^f^	Triggers Drug Interaction Cutoff	Fraction Inhibited^g^	Predicted Plasma AUC Ratio^h^	
									PDA	CP-I
Ritlecitinib										
Intestinal BCRP (RSV)	27 ± 2	2803	NA^a^	NA	NA	104	Yes	NA	NA	NA
Renal OAT1 (PAH)	156 ± 6	NA	4.7	NA	NA	0.03	Yes	0.03	1.1	NA
Renal OAT3 (E3S)	41 ± 3	NA	4.7	NA	NA	0.11	Yes	0.1		
Hepatic OATP1B1 (RSV)	312 ± 19	NA	NA	31.7	1.10	NA	Yes	0.09	ND^a^	ND
Hepatic OATP1B3 (RSV)	934 ± 59	NA	NA	31.7	1.03	NA	No	0.03		
Hepatic OATP1B1 (CP-I)	257 ± 8	NA	NA	31.7	1.12	NA	Yes	0.1	NA	1.1
Hepatic OATP1B3 (CP-I)	> 1000 (23%)^i^	NA	NA	31.7	< 1.03	NA	No	< 0.03		
M2										
Renal OAT1 (PAH)	> 300 (40%)	NA	1.4	NA	NA	< 0.01	No	< 0.01	ND	NA
Renal OAT3 (E3S)	> 300 (43%)	NA	1.4	NA	NA	< 0.01	No	< 0.01		
Hepatic OATP1B1 (RSV)	2.0 ± 0.03	NA	1.4^j^	NA	NA	0.70	Yes	0.4	NA	ND
Hepatic OATP1B3 (RSV)	8.4 ± 0.2	NA	1.4	NA	NA	0.17	Yes	0.1		

^a^Organic anion transporter (OAT), organic anion transporting polypeptide (OATP), *p*-aminohippuric acid (PAH), estrone 3-sulfate (E3S), rosuvastatin (RSV), coproporphyrin I (CP-I), breast cancer resistance protein (BCRP). NA, not applicable; ND, not determined

^b^Concentration of ritlecitinib and M2 presenting 50% inhibition of transporter in vitro (IC_50_). Data presented as mean ± SD of *n* = 3 replicates. For each transporter, the IC_50_ was determined at a low substrate concentration (PAH, E3S, RSV-OATP, CP-I, RSV-BCRP = 0.5, 0.1, 0.3–0.5, 0.1 and 0.2 µM, respectively) *versus* the reported Michaelis constant K_m_ (PAH = 5.0 µM, E3S = 9.5 µM, RSV OATP1B1 = 3.8–8.9 µM, RSV OATP1B3 = 28.3 µM, CP-I OATP1B1 = 1.4 µM, CP-I OATP1B3 = 14.4 µM, and RSV BCRP = 3.2 µM). Therefore, the IC_50_ approximates the inhibition constant (K_i_) and supports determination of fraction inhibited [14].

^c^C_max,u_ = maximal free plasma concentration (C_max_ × f_u_) of ritlecitinib (5.5 µM × 0.86) at 200 mg. [I]_2_ = oral molar dose of ritlecitinib/250 mL (molecular weight = 285.34)

^d^Etimated maximal free hepatic portal concentration of ritlecitinib I_max,inlet,u_ = f_u_ × [C_max_ + (F_a_ × F_g_ × *k*_a_ × Dose)/Q_h_/Rb], where maximal plasma concentration (C_max_) = 5.5 µM, fraction absorbed × fraction surviving gut first pass (F_a_ × F_g_) = 0.89, absorption rate constant (*k*_a_) = 7.9 h^−1^, liver blood flow (Q_h_) = 97 L/hr, blood-to-plasma ratio (Rb) = 1.62, and fraction unbound in plasma (f_u_) = 0.86 [10].

^e^*R*-value = 1 + (I_max,inlet,u_/IC_50_). Drug interaction risk cutoff *R*-value = 1.1 (US Food & Drug Administration) and 1.04 (European Medicines Agency) [14].

^f^Drug interaction risk cutoff C_max_/IC_50_ ratio = 0.1 (US Food & Drug Administration) and 0.02 (European Medicines Agency). [I]_2_/IC_50_ ratio (cutoff = 10) only applies to BCRP [15].

^g^Fraction inhibited calculated as described using Eqs. 1 and 2 below [14].

^h^CP-I and pyridoxic acid (PDA) plasma AUC ratio [AUC_(ritlecitinib)_/AUC_(reference)_] prediction as described in Table S1 and S2. For PDA, no substrate-dependency in IC_50_ is assumed

^i^Represents % inhibition at the highest concentration tested (1000 µM)

^j^C_max,u_ used for metabolite M2 and based on C_max_ and f_u_ of 1.5 µM and 0.95, respectively (Eq. 3). Rationale for using C_max,u_ for liver OATP1B1/3 is supported by the good absorption and low first pass metabolism of parent ritlecitinib (F_a_ × F_g_ = 0.89)

$$\frac{Imax,\;inlet,u}{Imax,\;inlet,u+{IC}_{50}}=Fraction\;Inhibited\;OATP\;(Eq.\;1)$$  $$\frac{Cmax,u}{Cmax,u+{IC}_{50}}=Fraction\;Inhibited\;OAT\;(Eq.\;2)$$  $$\frac{Cmax,u}{Cmax,u+{IC}_{50}}=Fraction\;Inhibited\;OATP\;for\;M2\;(Eq.\;3)$$

A modeling exercise was performed to predict an *in vivo* biomarker AUC response following ritlecitinib administration. Specifically, a static model was used to predict the plasma AUC ratio [AUC_(ritlecitinib)_/AUC_(reference)_] (AUCR) of PDA by leveraging existing *in vitro* inhibition data and C_max,u_ values for probenecid, a second Pfizer compound (PFE4), and pyrimethamine (Table S1, Figure S1) [14]. As shown in Table I, it was predicted that ritlecitinib would have a minimal impact on OAT1/3 *in vivo* by presenting a PDA AUCR of 1.1. Likewise, published data for ten different OATP1B1/3 inhibitors and rifampicin (assuming an absorption rate constant ka = 0.1 min^−1^ comparable to ritlecitinib) were used to validate a static model for plasma CP-I (Table S2, Figure S1). Similar to the case of OAT1/3, ritlecitinib was predicted to be a weak OATP1B1/3 inhibitor *in vivo* and presented a CP-I AUCR of 1.1 (Table I). Therefore, although ritlecitinib exceeded the regulatory OAT1/3 and OATP1B1 drug interaction risk cutoffs, it was anticipated that ritlecitinib would have a minimal impact on the AUC of both PDA and CP-I.

### Clinical Study

Twelve participants were assigned to treatment; all were treated and completed the study. Of the 12 participants, 10 were men and 2 were women; 10 were White, 1 Black, and 1 did not report race (Table II). The mean (SD) age was 38.7 (9.3) years with the range between 24 and 55 years.

**Table II:** Participant Demographics

	All participants (*N* = 12)
Sex, n (%)	
Male	10 (83.3)
Female	2 (16.7)
Race, n (%)	
White	10 (83.3)
Black or African American	1 (8.3)
Not reported	1 (8.3)
Ethnicity, n (%)	
Hispanic or Latino	3 (25.0)
Not Hispanic or Latino	9 (75.0)
Age (years)	
Median (range)	40.5 (24–55)
Mean (SD)	38.7 (9.3)
Weight (kg)	
Median (range)	74.9 (62.0 – 88.5)
Mean (SD)	73.6 (9.3)
Body mass index (kg/m^2^)	
Median (range)	23.1 (20.2–28.3)
Mean (SD)	23.6 (2.6)

For ABCG2 genotyping, one participant was heterozygous for SNP rs2231142 and all the others (*n* = 11) were wild-type. Based on the ABCG2 genotypes, all 12 participants are predicted to have a normal functioning BCRP protein. For SLCO1B1 genotyping, 7 participants had a genotype containing either *1A or *1B that is associated with a normal functioning OATP1B1. Five participants had one copy of the rs4149056 SNP, *1A/*15 or *1B/*5, which is associated with a decreased function of OATP1B1.

#### Plasma and Urine Rosuvastatin PK

**Fig. 2:**
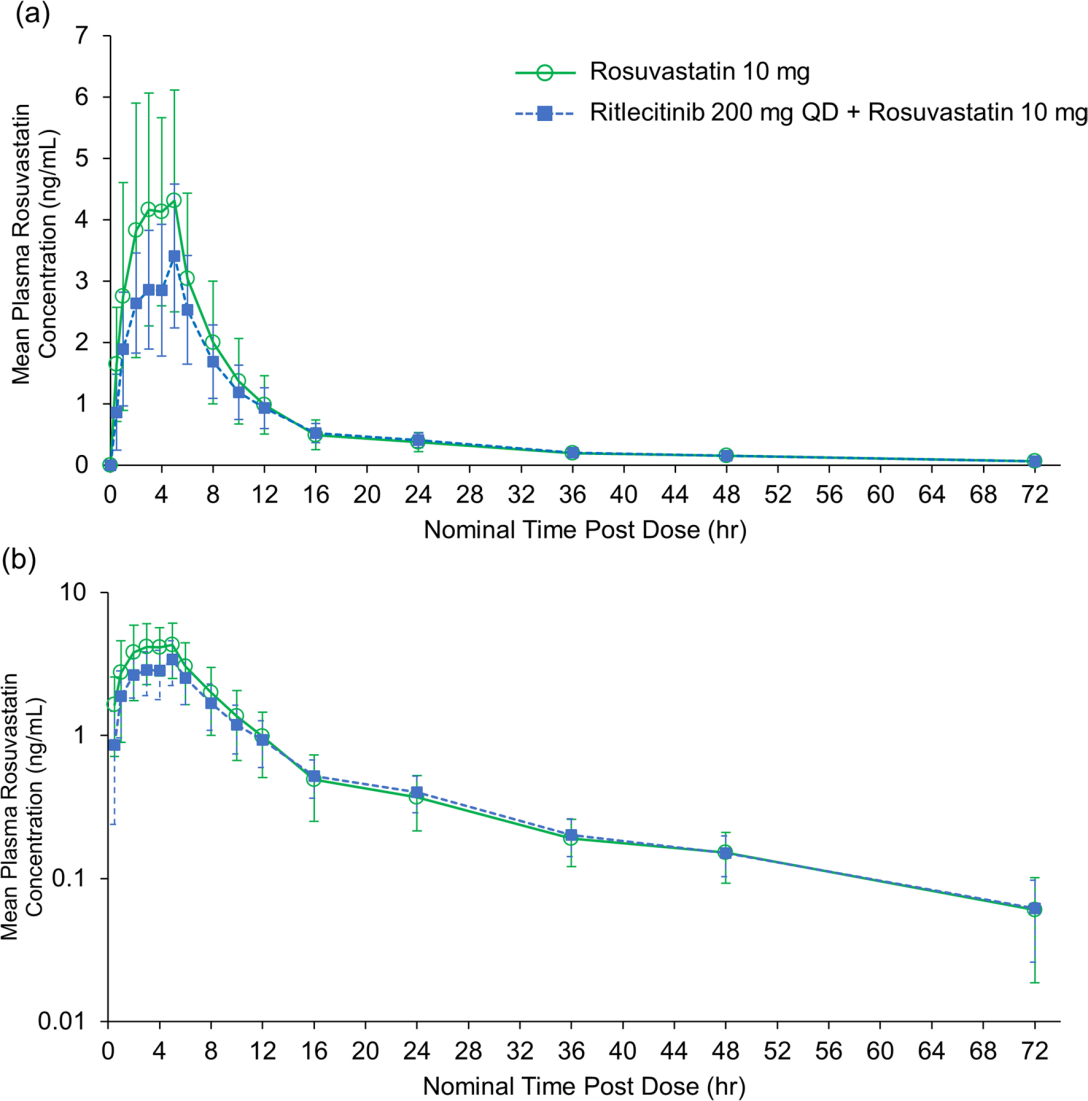
Mean plasma rosuvastatin concentration-time profiles following a single dose of rosuvastatin (10 mg) alone and with ritlecitinib (200 mg QD) coadministration in (**a**) linear scale and (**b**) semi-log scale. Error bars show the standard deviation. QD, once-daily.

Mean plasma rosuvastatin concentration-time profiles in the absence or presence of ritlecitinib coadministration are presented in Fig. 2. Plasma rosuvastatin concentration-time profiles were similar in the elimination phase with or without ritlecitinib coadministration and slightly lower in the absorption phase with ritlecitinib coadministration. Overall, rosuvastatin plasma exposure (AUC_inf_) and maximum plasma concentration (C_max_) decreased by  ~ 13% and ~ 27%, respectively, when coadministered with ritlecitinib (Table III). The median time to reach C_max_ (T_max_) was 4.01 h (range 2.00–5.03 h) when rosuvastatin was administered alone and 5.00 h (range 1.00–5.02 h) when coadministered with ritlecitinib (Table III). Mean elimination half-life (t_1/2_) for rosuvastatin was similar when administered alone (19.25 h) or with ritlecitinib (17.01 h) (Table III). The apparent oral clearance of rosuvastatin was slightly higher when rosuvastatin was coadministered with ritlecitinib than when it was administered alone (Table III).

**Table III:** Statistical Summary of Rosuvastatin Pharmacokinetic Parameters without and with Ritlecitinib coadministration

Parameter (Unit)*	Rosuvastatin 10 mg (*N* = 12)	Rosuvastatin 10 mg + Ritlecitinib 200 mg QD (*N* = 12)	% Ratio (Test/Reference) of Adjusted Geometric Means (90% CI)
Plasma			
AUC_inf_ (ng·hr/mL)	43.86 (44)	38.09 (34)	86.86 (74.91, 100.71)
C_max_ (ng/mL)	4.496 (43)	3.264 (39)	72.58 (63.25, 83.30)
CL/F (L/hr)	228.0 (44)	262.4 (34)	
t_1/2_ (hr)	19.25 ± 7.0220	17.01 ± 3.0402	
T_max_ (hr)	4.01 (2.00–5.03)	5.00 (1.00–5.02)	
V_z_/F (L)	6025 (63)	6346 (38)	
Urine			
Ae_72_ (mg)	0.5479 (46)	0.5059 (31)	
Ae_72_%	5.479 (46)	5.059 (31)	
CL_r_ (L/hr)	13.06 (21)	13.78 (13)	105.52 (98.52, 113.02)

*Geometric mean (%CV) for all, except median (range) for T_max_ and arithmetic mean ± SD for t_1/2_

AUC_inf_, area under the plasma concentration-time profile from time 0 extrapolated to infinite time; C_max_, maximum plasma concentration; CL/F, apparent oral clearance; t_1/2_, terminal plasma half-life; T_max_, time for C_max_; V_z_/F, apparent volume of distribution; Ae_72_, amount of drug excreted unchanged in urine up to 72 h; Ae_72_%, percent of dose excreted in urine; CL_r_, renal clearance; QD, once daily

Urine recovery of rosuvastatin was low, with  < 5.5% of the dose recovered unchanged in urine for both when administered alone and when coadministered with ritlecitinib (Table III). Renal clearance was comparable, with mean values of 13.06 L/hr when rosuvastatin was administered alone and 13.78 L/hr when coadministered with ritlecitinib.

#### Plasma PK of PDA and CP-I

**Fig. 3:**
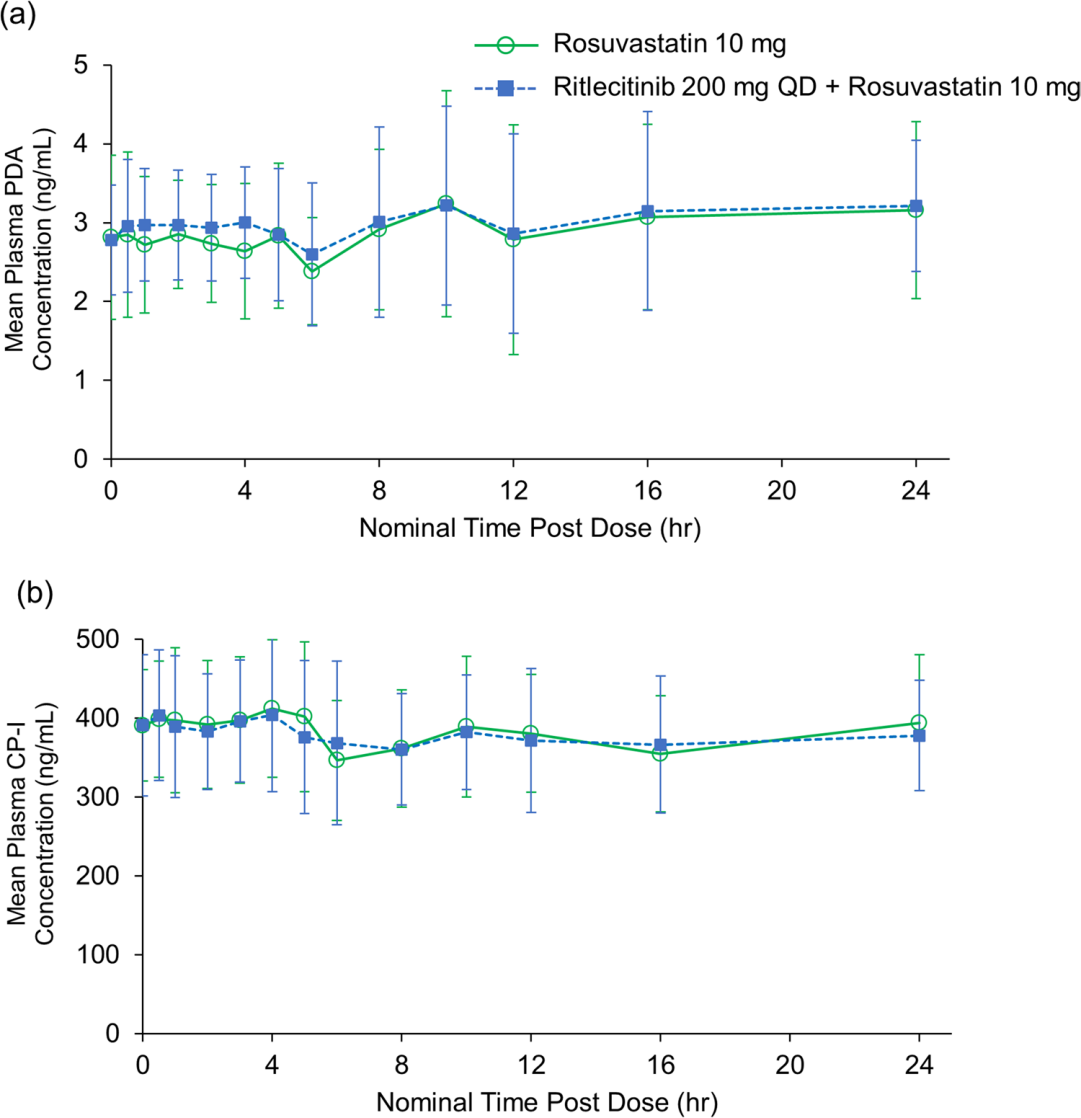
Plasma concentration-time profiles for (**a**) PDA and (**b**) CP-I following a single dose of rosuvastatin (10 mg) alone and with ritlecitinib (200 mg QD) coadministration. Error bars show the standard deviation. QD, once daily.

Mean plasma concentration time profiles were similar for both endogenous biomarkers PDA and CP-I with and without ritlecitinib coadministration (Fig. 3). Overall, PK parameters of PDA and CP-I, AUC_24_ and C_max_, were comparable in the presence and absence of ritlecitinib coadministration, with geometric mean ratios of 98%–103% (Table IV).

**Table IV:** Pharmacokinetic Parameters of PDA and CP-I with and without Ritlecitinib Coadministration

Parameter (Unit)*	Rosuvastatin 10 mg	Rosuvastatin 10 mg + Ritlecitinib 200 mg QD	% Ratio (Test/Reference) of Adjusted Geometric Means (90% CI)
PDA			
AUC_24_	67.34 (30)	69.47 (33)	103.16 (87.65, 121.42)
C_max_	3.664 (29)	3.765 (28)	102.76 (87.58, 120.58)
CP-I			
AUC_24_	8915 (21)	8729 (21)	97.91 (92.24, 103.94)
C_max_	427.1 (22)	418.8 (19)	98.06 (93.13, 103.26)

*Geometric mean (%CV) for all, except median (range) for T_max_

AUC_24_, area under the plasma concentration-time profile from time 0 to 24 h postdose; C_max_, maximum plasma concentration; CP-I, coproporphyrin I; PDA, pyridoxic acid; QD, once daily

#### Safety

A total of 13 treatment-emergent adverse events (TEAEs) were reported in this study. Of the 13 TEAEs, 4 occurred in 4 participants with the single dose of rosuvastatin 10 mg (without ritlecitinib), all of which were considered treatment-related by the investigator. The other 9 TEAEs occurred in 7 participants during treatment with ritlecitinib 200 mg QD, of which 7 TEAEs were considered treatment-related by the investigators. No TEAEs were reported for the coadministration of ritlecitinib 200 mg QD and single dose of rosuvastatin 10 mg. The most frequently reported TEAEs by system organ class were diarrhea (2 participants) and fatigue (2 participants). Twelve of the 13 TEAEs were mild in severity and 1 TEAE of back pain with the single dose of rosuvastatin 10 mg was moderate. There were no serious adverse events (AEs), severe AEs, dose reductions or discontinuations due to AEs.

## Discussion

As described herein, *in vitro* studies presented ritlecitinib as an inhibitor of BCRP, OATP1B1 and OAT1/3 based on standard regulatory DDI risk cutoffs, which triggered a dedicated clinical DDI study with rosuvastatin as a substrate drug for these transporters. However, in the subsequent clinical DDI study with rosuvastatin, rosuvastatin exposure was not increased and renal clearance was not decreased in the presence of ritlecitinib coadministration. Additionally, the presence of ritlecitinib did not impact CP-I and PDA exposures. Altogether, these data suggest that ritlecitinib does not inhibit BCRP, OATP1B1 and OAT3.

For a clinical DDI study where transporter inhibition risk is evaluated, single dose administration of the perpetrator is typically considered acceptable unless there is a time-dependent inhibition concern. In this study, multiple dose administration of ritlecitinib 200 mg QD was used (Fig. 1) because of the time-dependent PK characteristics observed with ritlecitinib. Ritlecitinib has a short half-life (arithmetic mean of 1.75 h for 200 mg single dose in first-in-human [FIH] study), thus accumulation was not expected for QD regimens. However, FIH study indicated steady-state of ritlecitinib PK was reached at Day 4 for the QD regimens and steady-state accumulation ratio of AUC was 1.8 for 200 mg QD regimen (data not published). Ritlecitinib is a moderate inhibitor of CYP3A and CYP1A2 enzymes, both of which are involved in metabolism of ritlecitinib (contribution of each pathway  < 25%, data not published). Therefore, mild time-dependent changes in PK may be expected due to this autoinhibition of drug metabolizing enzymes. To account for the time-dependent PK, 7 days of ritlecitinib QD dosing before rosuvastatin administration was implemented and ritlecitinib effect on rosuvastatin PK was evaluated at the steady-state level as a worst-case scenario. Even in this worst-case scenario, the study results demonstrated that ritlecitinib is not a clinical inhibitor of BCRP, OATP1B1 and OAT3.

Rosuvastatin was selected as a probe drug for BCRP, OATP1B1 and OAT1/3 transporters to evaluate perpetrator risk of ritlecitinib. Rosuvastatin is not extensively metabolized and is primarily excreted unchanged in feces via biliary excretion, with  ≤ 10% being excreted in urine [18, 19]. Several transporters are involved in absorption and excretion of rosuvastatin. BCRP is expressed in the small intestine and liver, and intestinal BCRP limits intestinal absorption of rosuvastatin as an efflux transporter, while liver BCRP determines biliary clearance of rosuvastatin by transporting rosuvastatin from hepatocyte into bile [20]. OATP1B1 is expressed in the basolateral side of hepatocytes and is involved in hepatic uptake of rosuvastatin and subsequent metabolism and elimination [21]. Therefore, potential inhibition of BCRP or OATP1B1 by ritlecitinib is expected to increase rosuvastatin systemic exposure. For the renal clearance of rosuvastatin, active tubular secretion accounts for the majority of total renal clearance, which is primarily mediated by OAT3 [21]. Given that renal clearance is not a major clearance pathway of rosuvastatin, the potential inhibition of OAT3 by ritlecitinib may not translate into significant increases in plasma concentration. Therefore, the renal clearance of rosuvastatin was measured in the current study as a more sensitive endpoint for changes in OAT3 activity. In a non-human primates study, coadministration of probenecid (an OAT3 inhibitor) has been shown to significantly decrease the renal clearance of rosuvastatin [22].

Based on the above-described mechanisms, potential transporter inhibition by ritlecitinib would be expected to increase systemic exposure to rosuvastatin. This lack of selectivity for individual transporters has made the use of some probe drugs challenging [7]. In the current study, CP-I and PDA data were assessed as endogenous markers to deconvolve any potential change in rosuvastatin exposure. CP-I is a by-product of heme synthesis and primarily eliminated as unchanged in feces via biliary excretion [23]. Sensitivity of plasma CP-I to OATP1B1/3 inhibition has been demonstrated in clinical DDI studies involving rosuvastatin with rifampicin as perpetrator. For example, CP-I exposure (AUC_24_) was increased by 4.0-fold with coadministration of rifampicin, a potent OATP1B1/3 inhibitor, compared to when rosuvastatin was administered alone [24]. Because of its high selectivity and sensitivity to OATP1B1/3, CP-I has been considered as a promising biomarker. PDA is an end-product of vitamin B6 catabolism and is eliminated via the kidney, with the excretion being enhanced by active tubular secretion [25]. Recent studies demonstrated that PDA is a substrate of OAT3 and its plasma exposure is sensitive to OAT3 inhibition such that AUC is increased by 3.1-fold in the presence of probenecid, a potent OAT3 inhibitor [26, 27]. Therefore, PDA data was measured in the study to investigate its utility as an endogenous biomarker for OAT3 by comparing its potential impact with that of the renal clearance of rosuvastatin. In the current study, neither an increase in rosuvastatin plasma exposure nor a decrease in rosuvastatin renal clearance was observed with ritlecitinib coadministration, indicating that ritlecitinib does not inhibit BCRP, OATP1B1 and OAT3. Lack of OATP1B1 and OAT3 inhibition potential for ritlecitinib was further supported by endogenous biomarker data of CP-I and PDA, as no changes of their plasma exposure were observed. Although no increase in rosuvastatin exposures was observed in the current study, the concurrent assessment of biomarkers CP-I and PDA would have provided a viable way to deconvolve which transporter is affecting the change if a clinically relevant interaction was observed.

However, a slight decrease in rosuvastatin plasma exposure was observed in the study, which cannot be explained by BCRP, OATP1B1 or OAT3 transporter inhibition. Similar plasma concentration-time profile in the elimination phase (Fig. 2) and similar T_max_ and t_1/2_ in the presence and absence of ritlecitinib coadministration (Table III) indicates that the change is likely due to a reduction in the absorption and/or enhanced first-pass elimination of rosuvastatin, and not due to a change in its elimination. Therefore, there may be another intestinal transporter driving such a change in absorption. Rosuvastatin is known to be a substrate of OATP2B1, which is expressed in small intestine and can enhance the intestinal absorption of substrate drugs as an influx transporter [28]. When rosuvastatin was coadministered with ronacaleret, an OATP2B1 inhibitor, rosuvastatin AUC_inf_ exposure was decreased by almost 50% without changes in T_max_ or t_1/2_ [29]. It can be hypothesized that potential inhibition of OATP2B1 by ritlecitinib may have resulted in the observed slight decrease in plasma exposure of rosuvastatin. In the previously reported sumatriptan DDI study, ritlecitinib coadministration decreased the C_max_ of sumatriptan by 13% even though AUC_inf_ of sumatriptan was increased by 30% due to OCT1 inhibition. The authors hypothesized the potential inhibition of another transporter, e.g., OATP2B1, by ritlecitinib to explain this differential impact [10]. Compared to other transporters, little is known about the clinical relevance of OATP2B1 and guidance for DDI evaluation for OATP2B1 is also lacking [30, 31]. Future work is needed to evaluate the OATP2B1 inhibition potential of ritlecitinib. Overall, the degree of change (13% reduction in AUC) is not considered to be clinically meaningful, thus rosuvastatin dose adjustment in the presence of ritlecitinib coadministration is not warranted [32].

It has been reported that the variability in rosuvastatin PK is associated with genetic polymorphisms of BCRP and OATP1B1. BCRP single-nucleotide polymorphism of 421C > A has been shown to increase rosuvastatin AUC by approximately twofold [33], and OATP1B1 polymorphisms of 388A > G and 521 T > C have been reported to affect variability in rosuvastatin AUC [34]. Thus, the magnitude of rosuvastatin exposure change in the presence of ritlecitinib could be limited in participants with these polymorphisms, which were investigated in this study. All 12 participants possessed the *ABCG2* genotype that is predicted to have normal BCRP function, *versus* 5 out of 12 participants had *SLCO1B1* genotype *1A/*15 or *1B/*5, either of which are predicted to have decreased OATP1B1 function. Further investigation on rosuvastatin PK parameters according to the *SLCO1B1* genotype indicated that the absence of an increasing effect on rosuvastatin exposure with ritlecitinib coadministration was similar across *SLCO1B1* genotype subgroups (Supplement Figure S2). Slightly higher rosuvastatin exposure was observed in the decreased OATP1B1 function subgroup (Supplement Figure S3), which is consistent with previous reports [34, 35].

It is noteworthy that ritlecitinib is one of the first compounds in development to assess drug transporter inhibition by leveraging four different endogenous biomarkers (PDA, CP-I, IBC, and NMN) integrated with a drug probe like rosuvastatin and sumatriptan [10]. Such an approach was warranted as ritlecitinib exceeded conservative regulatory DDI risk cutoffs for both renal (OAT1, OAT3, OCT 2, MATE1 and MATE2K) and hepatic (OCT1, OATP1B1, and OATP1B3) transporters. In addition, the possible impact of an inhibitory metabolite (M2) had to be addressed. As presented herein, it was possible to de-risk both ritlecitinib and its metabolite M2 as OAT1/3 and OATP1B1/3 inhibitors using plasma PDA and CP-I as biomarkers (AUCR ~ 1.0). A similar approach was leveraged to de-risk the inhibition potential of renal OCT2, MATE1, and MATE2K for ritlecitinib, because ritlecitinib administration did not inhibit the renal clearance of NMN in the previous study [10]. Based on drug probe and endogenous biomarker data from all completed transporter-mediated DDI studies, it has been demonstrated for both ritlecitinib and M2 that the inhibition of liver OCT1 is clinically evident (sumatriptan exposure increase by up to 50%) but differentiated from weak *in vitro* inhibition of OAT1/3, OATP1B1/3, OCT2, MATE1, and MATE2K. It is also worth noting that the minimal impact of ritlecitinib on rosuvastatin PK is consistent with other agents presenting a liver OATP1B1 *R*-value of 1.1 and intestinal BCRP *G*-value of  ~ 100 (Table I) [15]. The inclusion of PDA and CP-I in the present rosuvastatin-ritlecitinib interaction study would have supported the deconvolution of intestinal BCRP, liver OATP1B1/3, and renal OAT3 inhibition if an increase in rosuvastatin exposure had been observed. The results presented herein further showcase the utility of biomarkers to facilitate the generation of clinical inhibition data for transporters such as OAT1/3 (PDA) and OATP1B1/3 (CP-I), integrated with other biomarker data such as NMN (OCT2/MATEs) and IBC (OCT1), to enable the generation of a perpetrator pan-transporter inhibition signature. Furthermore, one can quickly address current regulatory DDI risk thresholds and de-risk false positive compounds, as well as support the deconvolution and mechanistic interpretation of probe drug PK data.

## Conclusions

Rosuvastatin plasma exposures as measured by AUC_inf_ and C_max_ were decreased by approximately 13% and 27%, respectively, in the presence of ritlecitinib coadministration, whilst renal clearance was comparable in the absence and presence of ritlecitinib coadministration. These DDI study results, in conjunction with plasma endogenous CP-I and PDA biomarker data, suggest that ritlecitinib does not inhibit BCRP, OATP1B1, and OAT3, despite an *in vitro* assessment surpassing the regulatory DDI risk cutoff of these transporters. Furthermore, our findings add to the body of evidence supporting the utility biomarkers to enable the generation of pan-transporter inhibition signatures as well as to guide a selection of dedicated clinical DDI studies without unnecessarily exposing healthy participants to drugs, and to support the mechanistic interpretation of complex DDI results for probe drugs.

### Supplementary Information

Below is the link to the electronic supplementary material.

Supplementary file1 (DOCX 398 KB)

## Data Availability

Upon request, and subject to review, Pfizer will provide the data that support the findings of this study. Subject to certain criteria, conditions, and exceptions, Pfizer may also provide access to the related individual de-identified participant data. See https://www.pfizer.com/science/clinical-trials/trial-data-and-results for more information.
