# Supplementary material for: Utilization of Rosuvastatin and Endogenous Biomarkers in Evaluating the Impact of Ritlecitinib on BCRP, OATP1B1, and OAT3 Transporter Activity
Source: Pharm Res. 2023 Aug 10;40(11):2639–51. doi: 10.1007/s11095-023-03564-3 (PMC10733197; doi:10.1007/s11095-023-03564-3)
Supplement: Supplementary file 1 — Supplementary file1 (DOCX 398 KB) [file 11095_2023_3564_MOESM1_ESM.docx]

**SUPPLEMENTAL MATERIAL**

**Supplemental Figure S1.** Predicting the impact of ritlecitinib on the plasma AUC of OAT1/3 (PDA) and OATP1B1/3 (CP-I) biomarkers.

**(A)** Observed versus predicted PDA AUC ratio [AUCR = AUC(inhibitor) /AUC(reference)] for ritlecitinib (RITLE), Pfizer compound 4 (PFE4), pyrimethamine at 3 dose (mg) levels (PYR) and probenecid (PROB). See Table S1 and fraction inhibition numbers for ritlecitinib in Table 1. Arrow indicates position of ritlecitinib. Dotted line is the line of identity (1:1 observed versus predicted). The shaded areas represent a PDA AUCR range of 0.8 to 1.25.

**(B)** Observed versus predicted change in plasma CP-I (ΔCP-I) as an AUC ratio [AUCR = AUC(inhibitor) /AUC(reference)] or change in a single CP-I concentration. Data for ritlecitinib (RITLE), rifampicin (RIF) and two series of perpetrator drugs are shown (see Table S2 and fraction inhibition numbers for ritlecitinib in Table 1). Arrow indicates position of ritlecitinib. Dotted line is the line of identity (1:1 observed versus predicted). The shaded areas represent a ΔCP-I range of 0.8 to 1.25.

**A
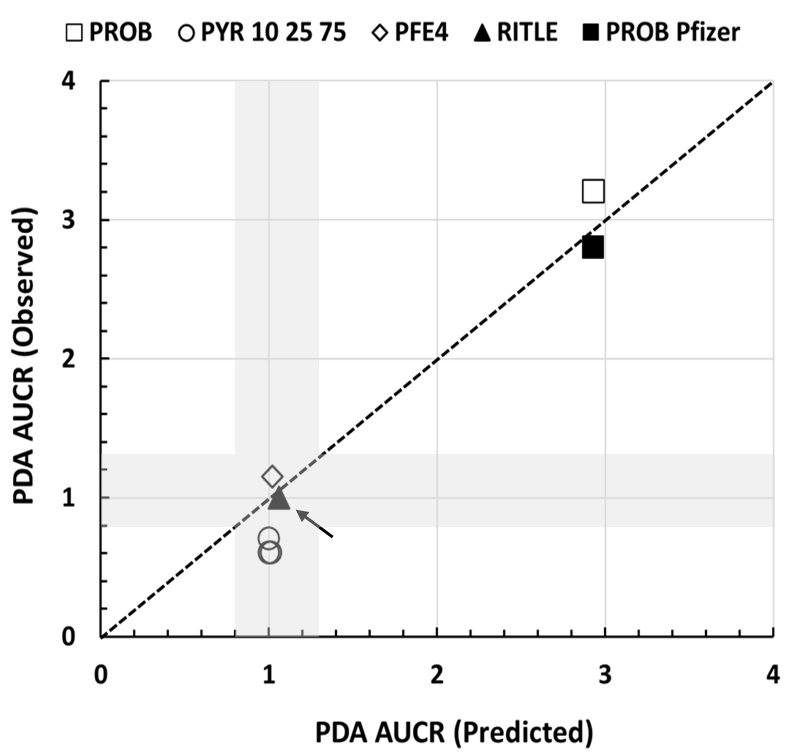
 B
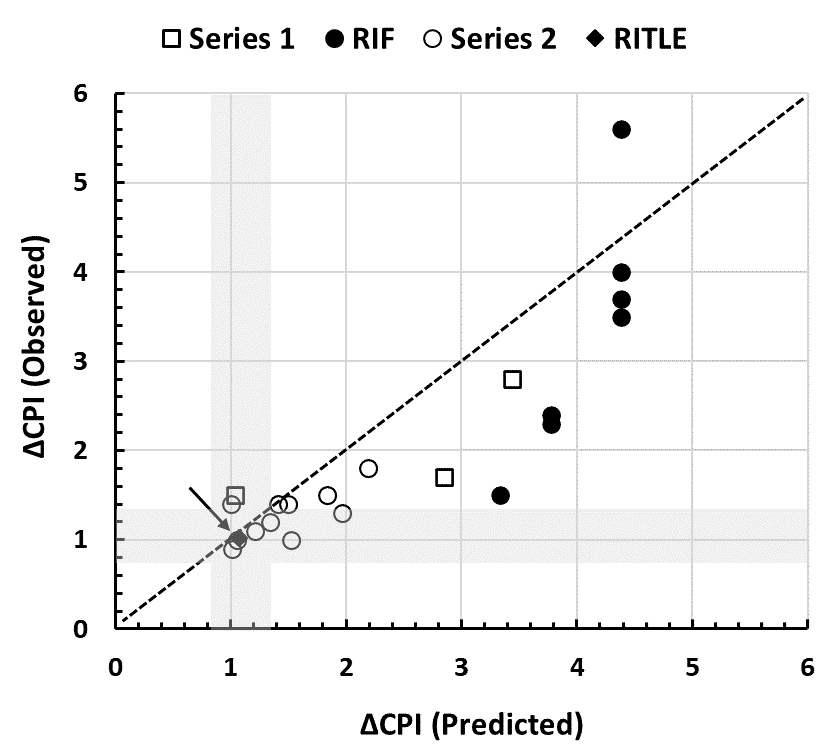
**

**Supplemental Table S1.** Predicting the plasma AUC ratio of the OAT1/3 biomarker PDA based on the in vitro IC_50_ for OAT1 and OAT3

| Inhibitor (mg) | C_max,u_  (µM)^a^ | IC_50_ (µM)^a^ | | ft_OAT3_^b^ | ft_OAT1_^b^ | AUC ratio^b^ | |
| --- | --- | --- | --- | --- | --- | --- | --- |
|  |  | OAT3 | OAT1 |  |  | Predicted^c^ | Observed |
| Probenecid 1000 | 22 | 4.3 | 13 | 0.66 | 0.17 | 2.9 | 3.2 |
| Probenecid 1000 (at Pfizer) | 22 | 4.3 | 13 | 0.66 | 0.17 | 2.9 | 2.8 |
| Pyrimethamine 10 | 0.04 | 16 | 224 | 0.66 | 0.17 | 1.2 | 0.7 |
| Pyrimethamine 25 | 0.11 | 16 | 224 | 0.66 | 0.17 | 1.2 | 0.6 |
| Pyrimethamine 75 | 0.37 | 16 | 224 | 0.66 | 0.17 | 1.0 | 0.6 |
| PFE4 65 | 0.07 | 2.9 | 2.4 | 0.66 | 0.17 | 1.0 | 1.1 |

AUC, area under the curve; C_max,u_, maximal free plasma concentration of inhibitor; OAT, organic anion transporter; PDA, pyridoxic acid.

^a^Concentration of inhibitor presenting a 50% decrease in activity (IC_50_) obtained at a final estrone 3-sulfate concentration of 0.1 µM (OAT3 K_m_ = 9.5 µM, Pfizer in-house data) and *p*-aminohippuric acid concentration of 0.5 µM (OAT1 K_m_ = 5.0 µM, Pfizer in-house data). Assumes no substrate-dependent shift in IC_50_ versus PDA.^1^ Each OAT form was singularly transfected in human embryonic kidney 293 cells.

^b^Rationale for the assigned fraction transported (ft) for each OAT has been described.^1^ References for observed AUCR are available.^1^

^c^AUC ratio = AUC(i)/AUC(c) predicted using the equation below based on determined fraction inhibited (FI) for each OAT.


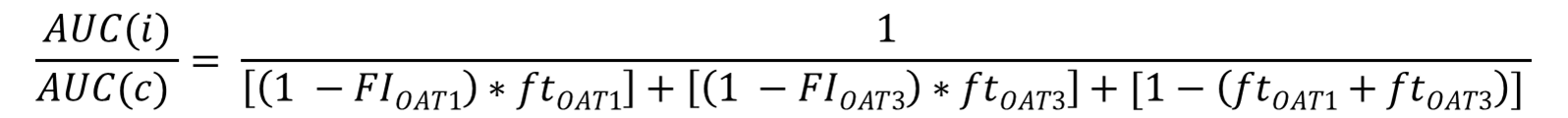


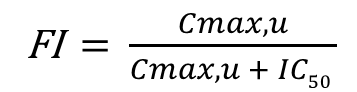


1. Rodrigues AD. Reimagining the framework supporting the static analysis of transporter drug interaction risk; integrated use of biomarkers to generate pan-transporter inhibition signatures. Clin Pharmacol Ther. 2023;113:986–1002. doi: 10.1002/cpt.2713.

**Supplemental Table S2.** Predicting the AUC ratio of OATP1B1/3 biomarker coproporphyrin isomer I (ΔCP-I) based on the *in vitro* IC_50_ for OATP1B1 and OATP1B3

| Series | Inhibitor (mg) | I_max,inlet,u_  (µM)^a^ | IC_50_ (µM)^a^ | | ft | | ∆CP-I | |
| --- | --- | --- | --- | --- | --- | --- | --- | --- |
|  |  |  | OATP1B1 | OATP1B3 | OATP1B1 | OATP1B3 | Predicted^c^ | Observed^b^ |
| Series 1 | Paclitaxel 200 | 1.5 | 0.2 | 0.7 | 0.67 | 0.18 | 3.4 | 2.8 |
|  | GDC-0810 600 | 0.009 | 0.2 | 0.9 | 0.67 | 0.18 | 1.0 | 1.5 |
|  | Cyclosporine A 100 | 0.4 | 0.2 | 0.05 | 0.67 | 0.18 | 2.8 | 1.7 |
| Rifampicin | Rifampicin 150 | 3 | 0.8 | 0.3 | 0.67 | 0.18 | 3.3 | 1.5 |
|  | Rifampicin 300 | 5.9 | 0.8 | 0.3 | 0.67 | 0.18 | 3.8 | 2.4 |
|  | Rifampicin 300 | 5.9 | 0.8 | 0.3 | 0.67 | 0.18 | 3.8 | 2.3 |
|  | Rifampicin 600 | 11.9 | 0.8 | 0.3 | 0.67 | 0.18 | 4.4 | 3.5 |
|  | Rifampicin 600 | 11.9 | 0.8 | 0.3 | 0.67 | 0.18 | 4.4 | 3.7 |
|  | Rifampicin 600 | 11.9 | 0.8 | 0.3 | 0.67 | 0.18 | 4.4 | 4 |
|  | Rifampicin 600 | 11.9 | 0.8 | 0.3 | 0.67 | 0.18 | 4.4 | 5.6 |
| Series 2 | Pimodivir 600 | 0.4 | 0.5 | 1.1 | 0.67 | 0.18 | 1.5 | 1.4 |
|  | Simeprevir 150 | 0.002 | 1.0 | 0.5 | 0.67 | 0.18 | 1.0 | 1.4 |
|  | Fenebrutinib 200 | 4.2 | 6.1 | 1.2 | 0.67 | 0.18 | 1.5 | 1 |
|  | Itraconazole 200 | 0.3 | 20 | 20 | 0.67 | 0.18 | 1.0 | 0.9 |
|  | Probenecid 1,000 | 56.6 | 73 | 118 | 0.67 | 0.18 | 1.4 | 1.4 |
|  | Diltiazem 240 | 8.6 | 100 | 175 | 0.67 | 0.18 | 1.1 | 1 |
|  | PF-06835919 280 | 8.2 | 4.3 | 5.6 | 0.67 | 0.18 | 1.8 | 1.5 |
|  | PF-06835919 50 | 1.5 | 4.3 | 5.6 | 0.67 | 0.18 | 1.2 | 1.1 |
|  | PF-06835919 600 | 15.3 | 4.3 | 5.6 | 0.67 | 0.18 | 2.2 | 1.8 |
|  | PF-06835919 400 | 10.3 | 4.3 | 5.6 | 0.67 | 0.18 | 2.0 | 1.3 |
|  | PF-06835919 100 | 2.5 | 4.3 | 5.6 | 0.67 | 0.18 | 1.3 | 1.2 |

AUC, area under the curve; C_max,u_, maximal free plasma concentration of inhibitor; OAT, organic anion transporter; PDA, pyridoxic acid.

^a^Concentration of inhibitor presenting a 50% decrease in activity (IC_50_) determined following a 30-minute pre-incubation. Final CP-I concentration = 0.1 µM (OATP1B1 and OATP1B3 K_m_ = 1.4 and 14.4 µM, respectively). For each inhibitor, the estimated maximal free hepatic portal concentration (I_max,inlet,u_) is shown (estimated assuming an absorption rate constant *k*_a_ = 0.1 min^-1^).^1^ Each OATP form was singularly transfected in human embryonic kidney 293 cells.

^b^References for the observed AUC ratio or single CP-I concentration ratio ± perpetrator are available.^1^

^c^AUC ratio = AUC(i)/AUC(c)) predicted using the equation below based on determined fraction inhibited (FI) and assigned fraction transported (ft) for each OATP.^1^


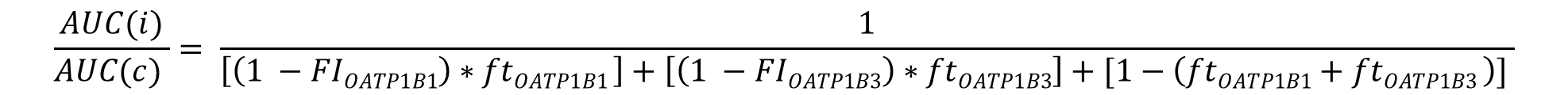


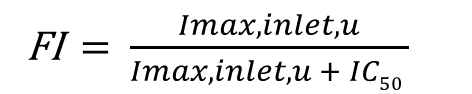


1. Rodrigues AD. Reimagining the framework supporting the static analysis of transporter drug interaction risk; integrated use of biomarkers to generate pan-transporter inhibition signatures. Clin Pharmacol Ther. 2023;113:986–1002. doi: 10.1002/cpt.2713.

**Supplemental Figure S2.** Comparison of (A) AUC ratio and (B) C_max_ ratio between test and reference group according to *SLCO1B1* genotype subgroups. AUC, area under the curve; C_max_, maximum plasma concentration.

**
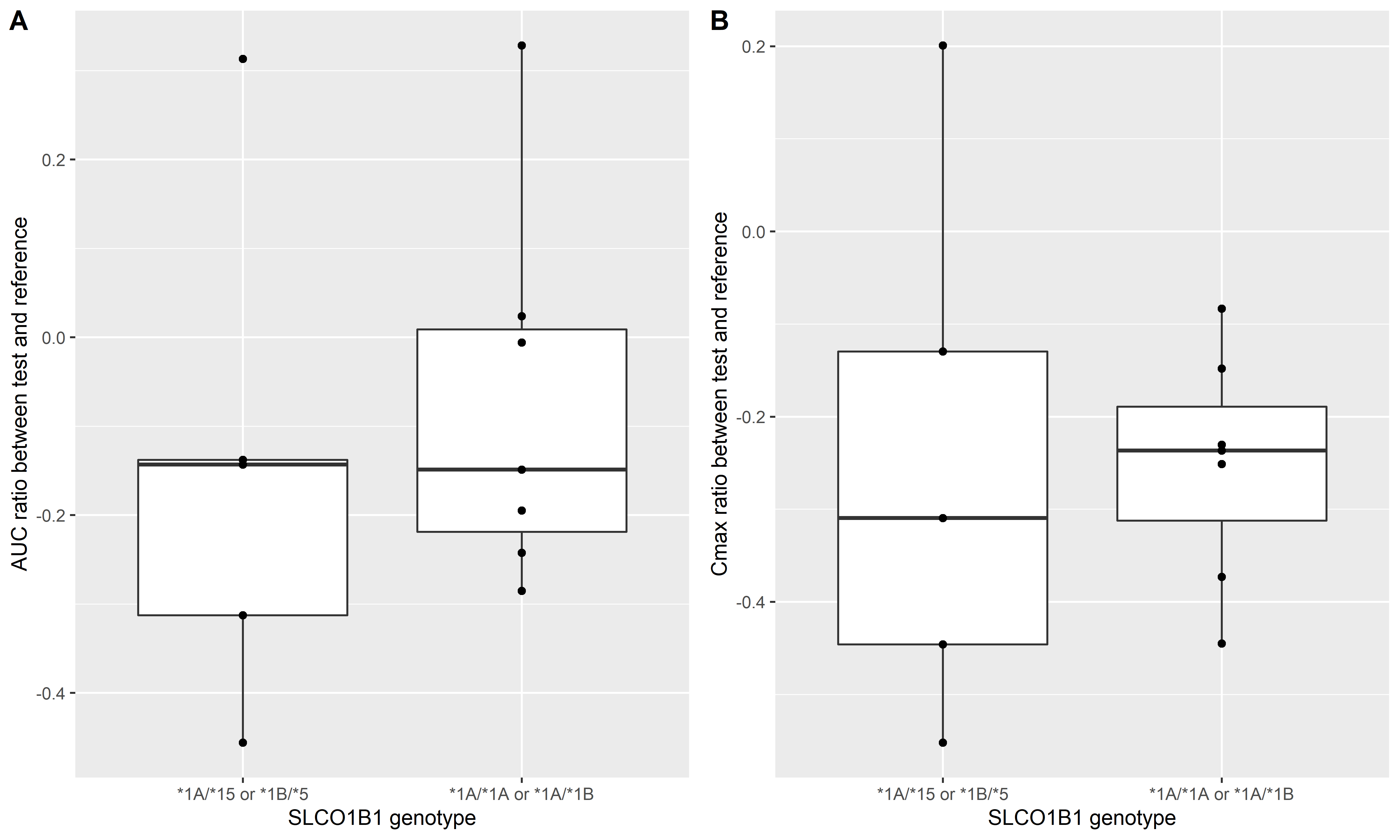
**

**Supplemental Figure S3.** Comparison of (A) AUC and (B) C_max_ of rosuvastatin (reference group only) according to *SLCO1B1* genotype subgroups. AUC, area under the curve; C_max_, maximum plasma concentration.

**
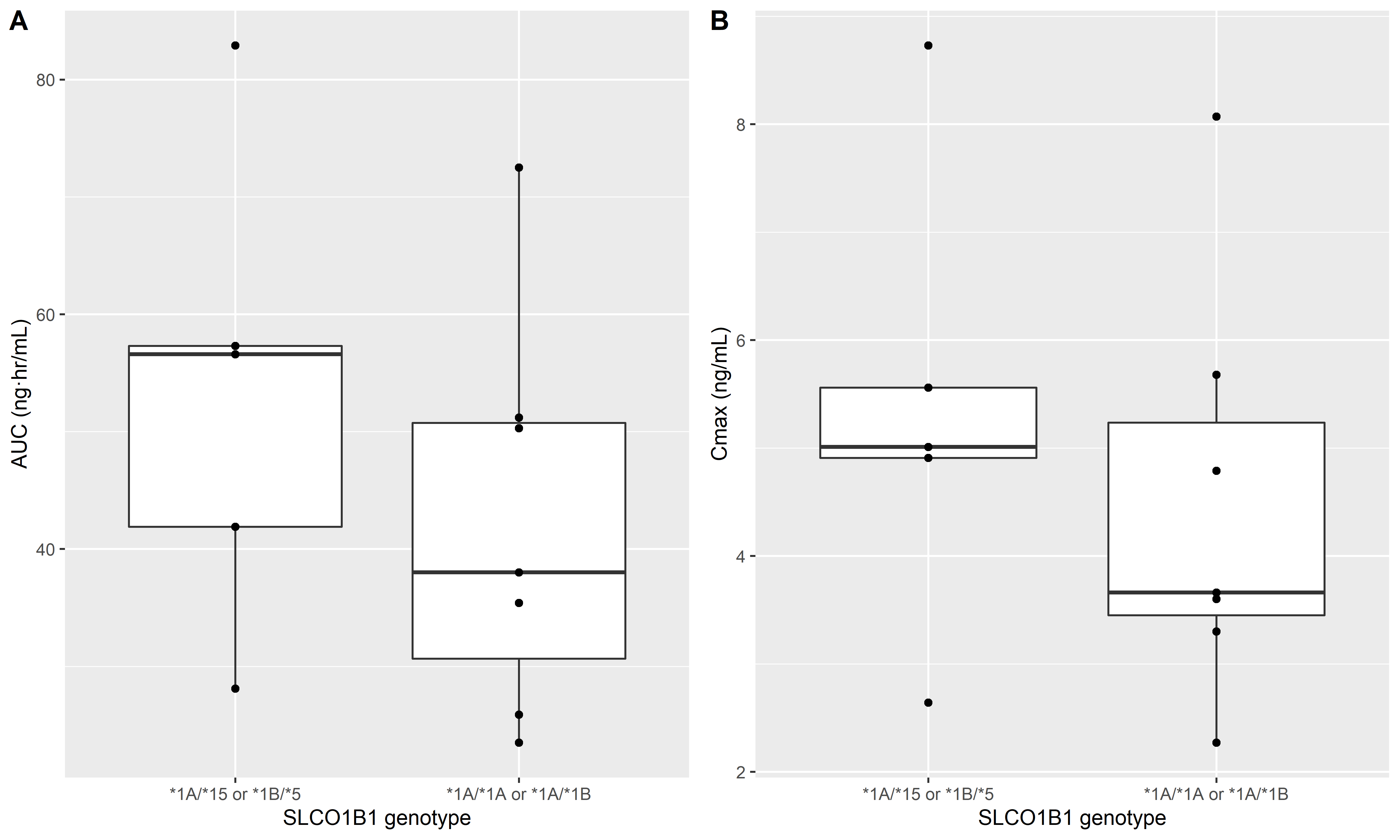
**
